# Prevention of mother-to-child transmission of hepatitis B virus: a phase III, placebo-controlled, double-blind, randomized clinical trial to assess the efficacy and safety of a short course of tenofovir disoproxil fumarate in women with hepatitis B virus e-antigen

**DOI:** 10.1186/s12879-016-1734-5

**Published:** 2016-08-09

**Authors:** Gonzague Jourdain, Nicole Ngo-Giang-Huong, Tim R. Cressey, Lei Hua, Linda Harrison, Camlin Tierney, Nicolas Salvadori, Luc Decker, Patrinee Traisathit, Wasna Sirirungsi, Woottichai Khamduang, Chureeratana Bowonwatanuwong, Thanyawee Puthanakit, George K. Siberry, Diane Heather Watts, Trudy V. Murphy, Jullapong Achalapong, Suchat Hongsiriwon, Virat Klinbuayaem, Satawat Thongsawat, Raymond T. Chung, Stanislas Pol, Nantasak Chotivanich

**Affiliations:** 1 Institut de recherche pour le développement (IRD, France), UMI 174 – PHPT, 187/10, Changklan Rd., Changklan, Muang, Chiang Mai 50100 Thailand; 2 Chiang Mai University, Faculty of Associated Medical Sciences, 110 Intawaroroj Rd., Sripoom, Chiang Mai 50200 Thailand; 3 Department of Immunology and Infectious Diseases, Harvard T.H. Chan School of Public Health, 677 Huntington Ave, Boston, MA 02115 USA; 4 Department of Molecular & Clinical Pharmacology, Institute of Translational Medicine, University of Liverpool, Liverpool, UK; 5 Center for Biostatistics in AIDS Research (CBAR), Harvard T.H. Chan School of Public Health, 677 Huntington Ave, Boston, MA 02115 USA; 6 Chonburi Hospital, 69 M.2, Sukhumvit Rd., Ban-suan, Muang, Chonburi 20000 Thailand; 7 Chulalongkorn University, Faculty of Medicine, 1873 Rama 4 Road, Pathumwan, Bangkok 10330 Thailand; 8 Eunice Kennedy Shriver National Institute of Child Health and Human Development, National Institutes of Health, 6100 Executive Blvd, Bethesda, MD 20892 USA; 9 US Department of State, Office of the Global AIDS Coordinator, SA-22, 2201 C Street NW, Washington, DC 20522-2210 USA; 10 Centers for Disease Control and Prevention, DHHS/CDC//NCHHSTP/DVH/Vaccine Unit Bldg Corporate SQ 12, Room 3111, Atlanta, GA 30329-1902 USA; 11 Chiangrai Prachanukroh Hospital, Obstetrics & Gynecology Department, 1039 Sathan Phayaban Rd., Muang, Chiang Rai 57000 Thailand; 12 Chonburi Hospital, Pediatrics Department, 69 M.2, Sukhumvit Rd., Ban-suan, Muang, Chonburi 20000 Thailand; 13 Sanpatong Hospital, Medical Department, 149 M.15 Yuhwa, Sanpatong, Chiang Mai 50120 Thailand; 14 Department of Statistics, Chiang Mai University, Faculty of Science, 239 Huaykaew Rd., Suthep, Muang, Chiang Mai 50200 Thailand; 15 Department of Internal Medicine, Chiang Mai University, Faculty of Medicine, Muang, Chiang Mai 50200 Thailand; 16 Massachusetts General Hospital, Gastrointestinal Unit, WRN 1007C, GI Unit, 55 Fruit St, Boston, MA 02114 USA; 17 Department of Hepato-Gastroenterology, Cochin University Hospital, 27 rue du Faubourg Saint-Jacques, 75679 Paris, Cedex 14 France

**Keywords:** Hepatitis B, Hepatitis B surface antigen, Hepatitis B e antigen, Pregnancy, Mother-to-child transmission, Thailand

## Abstract

**Background:**

Chronic hepatitis B virus (HBV) infection is complicated by cirrhosis and liver cancer. In Thailand, 6-7 % of adults are chronically infected with HBV. The risk of mother-to-child transmission (MTCT) of HBV has been estimated to be about 12 % when mothers have a high hepatitis B viral load, even if infants receive passive-active prophylaxis with HBV immunoglobulin (HBIg) and initiate the hepatitis B vaccine series at birth. We designed a study to assess the efficacy and safety of a short course of maternal tenofovir disoproxil fumarate (TDF) among women with a marker of high viral load for the prevention of MTCT of HBV.

**Methods:**

The study is a phase III, multicenter (17 sites in Thailand), placebo-controlled, double-blind, randomized 1:1, two-arm clinical trial of TDF 300 mg once daily versus placebo among pregnant women from 28 weeks’ gestation through 2-month post-partum. All infants receive HBIg at birth, and a hepatitis B (HB) vaccination series according to Thai guidelines: birth, and age 1, 2, 4 and 6 months. Participant women at study entry must be age ≥18 years, hepatitis B surface antigen (HBsAg) and e-antigen (HBeAg) positive, have alanine aminotransferase (ALT) level < 30 IU/L at screening (confirmed < 60 IU/L pre-entry), negative hepatitis C serology, creatinine clearance >50 mL/min, and no history of anti-HBV antiviral treatment.

The target sample size of 328 mother/infant pairs assumed 156 evaluable cases per arm to detect a ≥9 % difference in MTCT transmission (3 % experimental arm versus 12 % placebo arm) with 90 % power. Mothers and infants are followed until 12 months after delivery. The primary infant endpoint is detection of HBsAg, confirmed by detection of HBV DNA at six months of age. Secondary endpoints are maternal and infant adverse events, acute exacerbations of maternal hepatitis B disease (ALT >300 IU/L, defined as a “flare”) following discontinuation of study treatment, infant HBV infection status and growth up to 12 months of age.

**Discussion:**

The results of this randomized trial will clarify the efficacy and safety of a short course of antiviral treatment to prevent mother-to-child transmission of HBV and inform international guidelines.

**Trial registration:**

ClinicalTrials.gov Identifier NCT01745822.

## Background

Chronic hepatitis B virus (HBV) infection affects an estimated 240 million persons worldwide, is complicated by cirrhosis of the liver and hepatocellular carcinoma, and causes 650,000 deaths annually [1]. HBV is most infectious in persons with high-level viremia (HBV load) or positive HBeAg, a marker of high-level viral replication. In countries with a high prevalence (>8 %) of HBsAg-positive persons, and before the implementation of universal immunization programs, most HBV infections were the result of early childhood household transmission or maternal-to-child transmission (MTCT). Compared to other age groups, infants have the highest rate (about 90 %) of HBV infection progressing to chronic HBV infection. MTCT is thought to occur primarily during birth/delivery, but the timing and predominant route of transmission is poorly understood.

Efficient HB vaccines have been available since the early 1980s. The World Health Organization (WHO) recommends universal infant immunization, regardless of maternal HBV infection status, starting at birth. This intervention has dramatically reduced the prevalence of HBV infection in children wherever it has been implemented. For infants born to HBV-infected mothers, concurrent administration of HB immunoglobulin (HBIg) to the neonate as soon as possible after birth for added passive prophylaxis incrementally reduces the risk of infection [2]. However, studies have reported that 8 % to 12 % of infants born to mothers with high-level viremia are infected despite prophylaxis with passive (HBIg) and active (HB vaccine) immunization starting at birth [3–5]. It is possible that in utero transmissions cannot be prevented by interventions initiated after birth. Additionally, HBV escape mutants to HB vaccine- or HBIg- may be responsible for some transmissions [6, 7].

Studies have suggested that antivirals inhibiting HBV replication such as lamivudine, tenofovir disoproxil fumarate (TDF), and telbivudine, prescribed to pregnant women with high HBV loads at the end of pregnancy and during the early postpartum period can reduce the risk of MTCT [3, 4, 8]. This scientific evidence has been weakened by methodological shortcomings in the studies conducted to date. A recent meta-analysis and review concluded that “most of the data [were] derived from cohort studies, which are subject to significant biases, especially selection bias,” and stressed the “absence of studies warranting high confidence” and “a paucity of randomized clinical trials” [9]. This led the American Association for the Study of Liver Diseases not to recommend but rather “suggest antiviral therapy to reduce the risk of perinatal transmission of hepatitis B in HBsAg-positive pregnant women with an HBV DNA level >200,000 IU/mL”, rating the Quality/Certainty of Evidence as “Low” and the Strength of Recommendation as “Conditional” [10]. Similarly, in 2015, the first WHO Hepatitis Guidelines did not recommend this approach for the sole purpose of preventing MTCT of HBV (quality of evidence rated as “Very low”), even though some “countries in Asia have adopted a policy of treating highly viremic pregnant mothers with lamivudine, telbivudine or tenofovir” [1]. Moreover, the safety of the approach has not been fully assessed, specifically the risk of hepatic disease exacerbation (flare) following postpartum discontinuation of antiviral treatment.

The protocol described here was designed to address essential questions about the efficacy and safety of providing antiviral therapy to pregnant women who have markers of high HBV load to prevent MTCT of HBV. Indeed recent guidelines and reviews still stress the need for well conducted randomized clinical trials with sufficient sample size [1, 9, 10]. This study is being conducted in Thailand where antiviral treatment of pregnant women has not been implemented.

We hypothesize that a short course of TDF compared to placebo will reduce the risk of MTCT of HBV among HBeAg positive women, −-likely through a decrease in HBV load and, possibly, through a prophylactic effect in the fetus in combination with passive-active prophylaxis at birth in the neonate--, and that only clinically insignificant maternal flares will occur with postpartum antiviral discontinuation.

## Methods

**Fig. 1:**
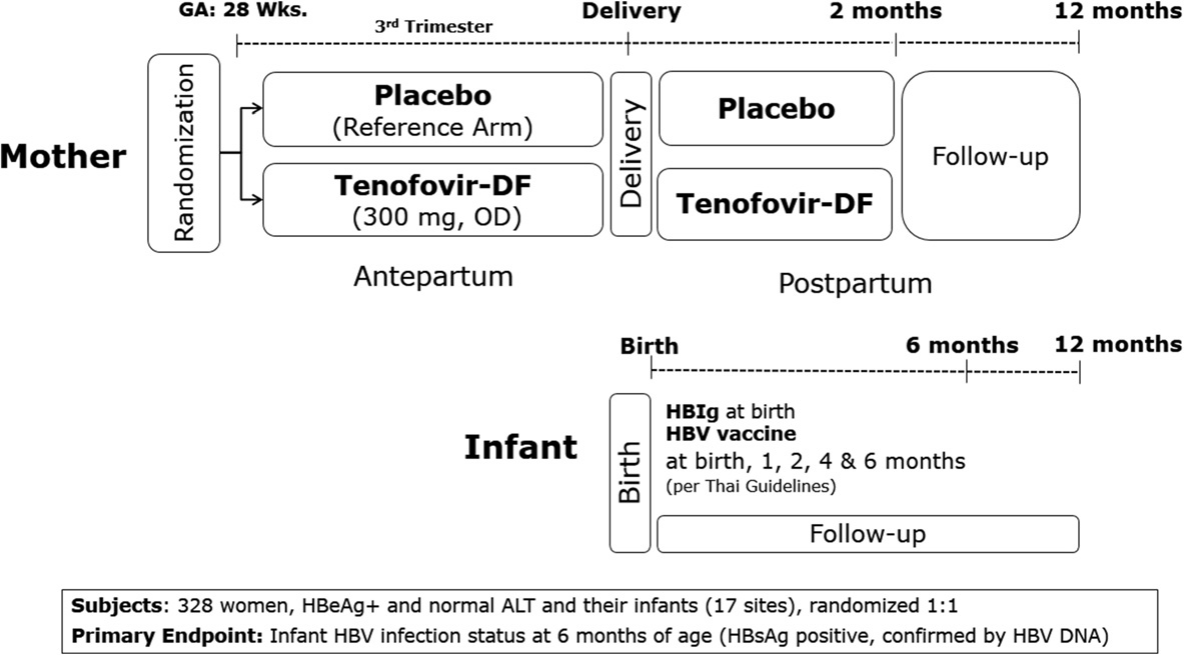
Study design

This is a phase III, multicenter, placebo-controlled, double-blind, randomized (1:1), two parallel arm clinical trial conducted in 17 public hospitals in Thailand (see Fig. 1). The list of locations is available at ClinicalTrials.gov Identifier NCT01745822. The primary objective is to assess the efficacy of TDF for the prevention of MTCT of HBV at 6 months of age. Important secondary objectives are to assess the risk of postpartum hepatic disease exacerbation after antiviral discontinuation, and infant growth through 12 months of age as part of the safety assessments.

Pregnant women are tested for the presence of HBsAg at their first antenatal care visit as standard of care in Thailand. At each site, all pregnant women aged 18 years or older with a positive HBsAg test were informed about the study and invited to provide their informed consent for participation.

The following study eligibility criteria are verified at enrollment:

Inclusion Criteria:

- Pregnancy
- At least 18 years of age
- Negative Human Immunodeficiency Virus serology
- Positive HBsAg and HBeAg tests
- Gestational age of 28 weeks (+ or - 10 days) as determined by the study site obstetrician
- Alanine aminotransferase (ALT) ≤30 IU/L, confirmed ≤60 IU/L on a subsequent blood draw
- Pregnant woman agrees to bring her infant at the planned study visits at a study site, until one year after delivery and to inform the site investigator if she plans to move to another place and not be able to return to the clinic.
- Pregnant woman understands the need for adequate infant immunization and agrees to the blood draws from her infant and the need for close follow-up to manage possible exacerbation of hepatitis.

Exclusion Criteria:

- History of TDF treatment at any time, or any other anti-HBV treatment during the current pregnancy.
- Creatinine clearance <50 mL/min, calculated using the Cockcroft-Gault formula.
- Dipstick proteinuria > 1+ (>30 mg/dL) or normoglycemic glucosuria confirmed on two separate occasions.
- Positive serology for Hepatitis C infection.
- Evidence of fetal anomalies incompatible with life.
- Any concomitant condition or treatment that, in the view of the clinical site investigator, would contraindicate participation or satisfactory follow-up in the study.
- Concurrent participation in any other clinical trial without written agreement of the two study teams.

At 28 weeks gestation (+/−10 days), eligible participants are enrolled and randomly assigned to receive the study treatment (manufactured by Gilead Sciences Inc.) of either TDF 300 mg once daily in the “TDF” experimental arm or a matching placebo in the reference arm, from 28 weeks gestation through 2-months postpartum. Women are instructed to return to the study pharmacist any unused study treatment and empty bottles; adherence to treatment is assessed by self-report and exact pill count. All infants receive passive-active prophylaxis with HBIg at birth and HB vaccination according to Thai guidelines: at birth, and ages 1, 2, 4 and 6 months of age. Following WHO guidelines, mothers are encouraged to breastfeed as there is no evidence of additional transmission during breastfeeding [11, 12].

Mothers and infants are followed until 12 months after delivery. Maternal study visits are conducted at 28, 32, 36 weeks’ gestation, delivery, and at 1, 2, 3, 4, 6 and 12 months postpartum. For mothers with confirmed moderate liver disease exacerbation (ALT >60 IU/L) after discontinuation of the study treatment, specific evaluations are conducted including bilirubin, prothrombin/International Normalized Ratio, and albumin, and plasma is stored for further retrospective evaluations. Additional investigations are performed for case management and documentation, and reintroduction of the double-blind study treatment is considered following a pre-defined algorithm by the reference site internist if high ALT levels persist. Infant visits are scheduled at birth, 1, 2, 4, 6, 9 and 12 months of age for clinical safety evaluation and determination of HBV status.

Serious adverse events (SAEs) that occur in either arm, as defined by 1996 International Conference on Harmonisation Good Clinical Practice, whether or not considered to be related to the study treatment by the study site investigator, are immediately reported to the Thai Ministry of Public Health and to the *Eunice Kennedy Shriver* National Institute of Child Health & Human Development (NICHD) and Centers for Disease Control and Prevention (CDC) program scientists, and those related to the study treatment to the drug manufacturer.

The primary endpoint is the detection of HBsAg that is confirmed by detectable HBV DNA in infants at six months of age. Secondary endpoints are infant’s HBV infection status, defined as detection of HBsAg confirmed by detectable HBV DNA at or after 6 months through 12 months of age, occurrence of maternal and infant adverse events (SAEs and grade 3/4 signs and symptoms in the Division of AIDS Table for Grading the Severity of Adult and Pediatric Adverse Events, Version 1.0, December, 2004 [13]), occurrence of flares (ALT >300 IU/L) following discontinuation of study treatment, and infant growth measured by weight, height and head-circumference at 6 and 12 months of age.

### Randomization, allocation concealment, and blinding

The sequentially numbered permuted blocks (of size undisclosed to the investigators) randomization list was computer generated by the study statistician (who had no further involvement in study treatment allocation) before the study started. The list, securely transmitted to the study pharmacist only, is used for secondary packaging of study treatment, consisting of 30-tablet bottles, into sequentially ordered packages. Stratification by site is achieved by shipping packages of study treatment divisible by the block size and blinding is achieved by only identifying study treatment by the patient identification number dictated by the sequential list. Patients, study staff on site and at the coordination center, investigators and laboratory personnel in charge of HBV related tests are blind to the randomized assignment.

### Sample size

It was calculated that a total of 312 evaluable mother-infant pairs were needed to detect a ≥9 % difference in HBV transmission between arms (3 % in the experimental arm versus 12 % in the placebo arm) with 90 % power using a Fisher’s exact test and a one-sided 0.049 significance level to account for one interim efficacy analysis. This difference is equivalent to a relative reduction of 75 % in HBV infection rate in the TDF arm. It was planned to enroll 328 pregnant women to account for an estimated 5 % loss to follow-up. If the infection rate in the placebo arm is only 10 %, the study has 83.2 % power to detect a 75 % reduction in the infection rate, i.e., an infection rate at 2.5 % in the TDF arm. However, if the infection rate is only 8 %, the power of detecting a 75 % reduction in the infection rate (2 % infection rate in the placebo arm) drops to 72.6 %.

### Monitoring

The composition of the Data and Safety Monitoring Board (DSMB) adhered to the following criteria: at least 1 gastroenterologist/hepatologist, 1 pediatrician, 1 obstetrician, 1 infectious diseases specialist, and 1 statistician/ epidemiologist, at least 3 of them from Thailand. Program scientists participate as observers in open sessions of DSMB calls/meetings. The study was presented to the DSMB during a protocol initiation review before study initiation. At annual meetings, the DSMB reviews the conduct of the study and adverse events. One interim review to monitor efficacy is planned when 50 % of the information on the primary endpoint is available, i.e. when 164 infants either have their 6-month HBV status available or are off study before the 6-month visit. A Haybittle-Peto function is employed for the group sequential design, so that at the interim efficacy review, the comparison of the primary endpoint is considered significant only if *p* < 0.001.

### Statistical analyses

For the primary objective/endpoint, the proportions of infants with positive HBsAg, confirmed by HBV DNA, at 6 months of age, along with their 95 % confidence intervals, will be provided by arm based on the exact Binomial distribution and compared between the TDF arm and the placebo arm based on Fisher’s exact test. The test will be adjusted for the interim efficacy monitoring by considering *p* < 0.05 – 0.001 = 0.049 as significant. If mothers’ baseline HBV DNA loads are imbalanced between treatment arms, an exact logistic regression will be conducted as a sensitivity analysis. The primary analysis will be conducted as a complete case analysis including infants whose 6-month endpoint is available in their original randomized group, regardless of the duration of study treatment their mothers actually received - if at least one dose was taken. A secondary analysis will be conducted in a modified intent to treat (mITT) population, where all randomized pregnant women who received at least one dose of study treatment will be included, and infants who are lost to follow-up before their 6 months HBV status is available will be considered as infected. Further sensitivity analysis may be performed, including imputing infants’ last available HBV infection status as their 6 months status for those who are lost to follow-up prior to 6 months or multiple imputations accounting for possible reasons of loss to follow-up, to explore the effect of loss to follow-up. A secondary supporting analysis will be conducted also considering as HBV infected infants with positive HBsAg, confirmed by HBV DNA, after 6 months and up to 12 months of age. Logistic regression, accounting for factors that are known to be associated with mother-to-child HBV transmission, could also be conducted to provide an adjusted odds ratio between treatment arms.

For secondary objectives/endpoints, proportions of women with severe hepatic disease exacerbation (ALT >300 IU/L, regardless of baseline values) occurring after study treatment discontinuation and up to 12 months postpartum will be compared between treatment arms by Fisher’s exact test. Proportions of women with any adverse event (any SAE or grade 3/4 sign or symptom defined by the Division of AIDS) from randomization until study exit will be analyzed similarly. Poisson regressions, with an offset of follow-up time will be further conducted to model repeated occurrence of adverse events. Adverse events in infants will be analyzed in a similar manner. Infants’ mean weight, height and head circumference z-scores at 6 and 12 months of age will be compared between arms based on normal approximation and two-sample t-tests.

In exploratory analysis, the proportion of infants with detectable 9 month HBV DNA and that of infants with seroprotection (anti-HBs antibodies > 10 IU/L) will be compared by treatment arm, as well as the proportions of premature labors (birth before 37 weeks of gestational age assessed by Ballard Score). Additional exploratory analyses include, but are not limited to, analysis on time to treatment discontinuation and/or study discontinuation and analysis on adherence.

Quarterly conference calls involving the core protocol team and program scientists are held to review the progress of the study and adverse events. The implementation of the study at each site is audited by a contract research organization one to three times during the study period, depending on the number of enrollees.

All site investigators have been trained in Good Clinical Practice and Human Subjects Protection. The trial protocol was reviewed and approved on September 3, 2012 by the Ethics Committees of the Institute for the Development of Human Research Protections at the Thailand’s Ministry of Public Health (reference สคม 1340/2555), the Faculty of Associated Medical Sciences, Chiang Mai University, on October 18, 2012 (reference 525/2555), the NICHD, and the CDC, as well as by the ethics committees of participating sites in Thailand.

## Discussion

This phase III randomized placebo-controlled double-blind trial will assess the efficacy and safety of a short course antiviral treatment to prevent mother-to-child transmission of HBV. Enrollment was opened in January 2013 and accrual was completed on August 19, 2015 with no significant operational issues. The data collection for the primary endpoint is scheduled to be completed in August 2016. Primary efficacy results are expected soon afterwards. The efficacy and safety study results will help clarify whether this strategy can play a significant role in the prevention of mother-to-child HBV transmission in highly viremic women.

## Abbreviations

ALT, alanine aminotransferase; CDC, centers for disease control and prevention; DSMB, data and safety monitoring board; HB, hepatitis B; HBeAg: hepatitis B e-antigen; HBIg, hepatitis B specific immunoglobulin; HBsAg, hepatitis B surface antigen; HBV, hepatitis B virus; IU, international units; mITT, modified intent to treat; MTCT, mother-to-child transmission; NICHD, Eunice Kennedy Shriver National Institute of Child Health & Human Development; SAE, Serious adverse events; TDF, tenofovir disoproxil fumarate; WHO, World Health Organization
